# Impact of pathobiological diversity of *Mycobacterium tuberculosis* on clinical features and lethal outcome of tuberculosis

**DOI:** 10.1186/s12866-022-02461-w

**Published:** 2022-02-08

**Authors:** Igor Mokrousov, Oksana Pasechnik, Anna Vyazovaya, Irina Yarusova, Alena Gerasimova, Aleksey Blokh, Viacheslav Zhuravlev

**Affiliations:** 1 Laboratory of Molecular Epidemiology and Evolutionary Genetics, St. Petersburg Pasteur Institute, 14 Mira street, St. Petersburg, 197101 Russia; 2 Department of Public Health, Omsk State Medical University, Omsk, Russia; 3 Bacteriology Laboratory, Clinical Tuberculosis Dispensary, Omsk, Russia; 4 Department of Epidemiology, Omsk State Medical University, Omsk, Russia; 5 St. Petersburg Research Institute of Phthisiopulmonology, St. Petersburg, Russia

**Keywords:** *Mycobacterium tuberculosis*, Beijing genotype, Lethal outcome, Multidrug resistance, Virulence

## Abstract

**Background:**

*Mycobacterium tuberculosis* population in Russia is dominated by the notorious Beijing genotype whose major variants are characterized by contrasting resistance and virulence properties. Here we studied how these strain features could impact the progression of pulmonary tuberculosis (TB) concerning clinical manifestation and lethal outcome.

**Results:**

The study sample included 548 M*. tuberculosis* isolates from 548 patients with newly diagnosed pulmonary TB in Omsk, West Siberia, Russia. Strains were subjected to drug susceptibility testing and genotyping to detect lineages, sublineages, and subtypes (within Beijing genotype). The Beijing genotype was detected in 370 (67.5%) of the studied strains. The strongest association with multidrug resistance (MDR) was found for epidemic cluster Beijing B0/W148 (modern sublineage) and two recently discovered MDR clusters 1071–32 and 14717–15 of the ancient Beijing sublineage. The group of patients infected with hypervirulent and highly lethal (in a mouse model) Beijing 14717–15 showed the highest rate of lethal outcome (58.3%) compared to Beijing B0/W148 (31.4%; *P* = 0.06), Beijing Central Asian/Russian (29.7%, *P* = 0.037), and non-Beijing (15.2%, *P* = 0.001). The 14717–15 cluster mostly included isolates from patients with infiltrative but not with fibrous-cavernous and disseminated TB. In contrast, a group infected with low virulent 1071–32-cluster had the highest rate of fibrous-cavernous TB, possibly reflecting the capacity of these strains for prolonged survival and chronicity of the TB process.

**Conclusions:**

The group of patients infected with hypervirulent and highly lethal in murine model 14717–15 cluster had the highest proportion of the lethal outcome (58.3%) compared to the groups infected with Beijing B0/W148 (31.4%) and non-Beijing (15.2%) isolates. This study carried out in the TB high-burden area highlights that not only drug resistance but also strain virulence should be considered in the implementation of personalized TB treatment.

**Supplementary Information:**

The online version contains supplementary material available at 10.1186/s12866-022-02461-w.

## Introduction

Recognition of the clinical significance of *Mycobacterium tuberculosis* population diversity is the key issue in molecular epidemiology and personalized medicine of tuberculosis (TB). Strains of different genetic lineages of *M. tuberculosis* demonstrate variability in some biological properties such as, in vitro growth rate, virulence in animal models, capacity to acquire drug resistance. *M. tuberculosis *sensu stricto is a clonal species with a hierarchical population structure that includes four major lineages L1 to L4 while other smaller lineages are mainly delimited to their areas of origin in Africa. An association of the genotype of some strains of lineages 2 and 4 with the severity of the clinical course of the disease and increased transmissibility was shown in different settings [1–3]. The outcome of the disease, favorable or adverse, depends on a number of factors that act independently or synergistically and include not only the strain virulence, but also human genetics, HIV coinfection, immunosuppression, duration of tuberculosis disease, the timeliness of diagnosis, treatment efficacy, and social and environmental factors [1, 3–7].

**Fig. 1:**
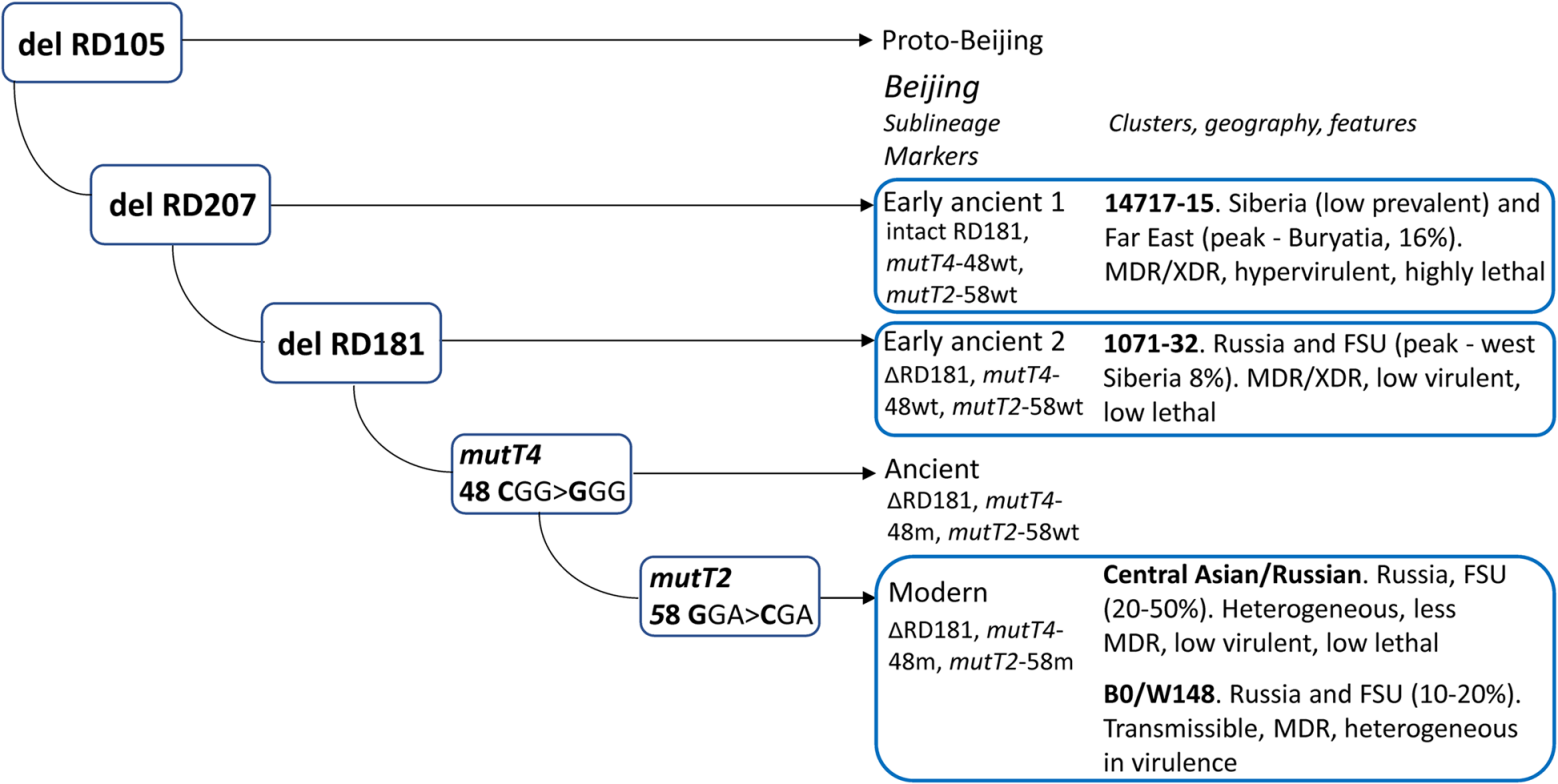
The simplified evolutionary pathway of the *M. tuberculosis* Beijing genotype with a focus on the genetic clusters analyzed in this study (highlighted in bold)

The Beijing genotype is the key member of the East Asian lineage (or Lineage 2) and is probably the most studied genetic family of *M. tuberculosis*. While multi/extensively-drug resistant (MDR/XDR) TB outbreaks caused by Beijing genotype were reported [8–10], the importance of precisely identifying particular strains or clonal clusters within this family became apparent for adequate epidemiological surveillance. Application of phylogenetic molecular markers along with high resolution genotyping methods permitted to gain a more informed view on the population structure of this genetic family. The Beijing genotype is subdivided into large-scale phylogenetic sublineages that are commonly termed as ancient/ancestral and modern. At the high-resolution level, genetic clusters of the closely related isolates have been identified and some of them have particular features of clinical significance, to begin with the W strain that caused the New York City outbreak in the mid-1990s [8]. In Russia, such important Beijing clusters are represented by two major and endemic genotypes B0/W148 (also termed as Russian epidemic/successful strain [11]) and Central Asian/Russian [12]. Both are widespread across all Russia and other countries of the Former Soviet Union but differ in association with drug resistance, B0/W148 being the most dangerous. Both types belong to the modern sublineage of the Beijing genotype that is otherwise dominant in Russia and most of the world. Bespyatykh et al. [13] demonstrated that major Russian clusters of the modern Beijing sublineage are heterogeneous in their virulence in the murine model and do not always demonstrate particularly high virulence (e.g. increased bacterial load in lungs) compared to the control strain H37Rv*.* Central Asian/Russian clade was less virulent and lethal in that study [13] and less frequently MDR compared to the B0/W148 strain in the other study [14]. The MDR-associated B0/W148 demonstrated variable virulence properties in different studies (reviewed in [3, 11, 13]) but its increased clustering and prevalence in the prison settings was interpreted as due to an increased capacity for transmission [11]. Ancient sublineage of the Beijing genotype includes genetically distant branches that bear common ancestral alleles in some loci (hence another name of this group as ancestral sublineage) and its strains are mainly drug-susceptible [15]. Until recently, due to their low prevalence in Russia, ancient Beijing sublineages received much less attention than dominant strains of the modern sublineage. We have recently discovered two clusters of the ancient Beijing sublineage circulating in the Asian part of Russia both of which were, to our surprise, strongly associated with MDR/XDR [16, 17]. The in vivo based study demonstrated their contrasting virulence and lethality properties in the mouse model [18]. The low virulent Beijing 1071–32-cluster is relatively widespread across the former Soviet Union but at low prevalence except for Omsk in western Siberia, where it accounted for 7% of the total population. In contrast, highly lethal and hypervirulent Beijing 14717–15 is endemically prevalent only in Buryatia, Far East but at quite a high rate (16%) in the total *M. tuberculosis* population [19]. The simplified evolutionary pathway of the Beijing genotype with information on the above subtypes is shown in Fig. 1.

The emergence of the unexpectedly highly resistant clusters highlights the importance of their surveillance and analysis in order to understand underlying reasons and consequences of their spread and impact on TB control programs. We hypothesized that the pathogenetic properties of strains can be manifested at the patient level in terms of clinical form of TB and lethal outcome. To this end, we analyzed strain and patient-related characteristics of the large *M. tuberculosis* dataset from the Omsk region in western Siberia.

## Materials and methods

Study collection included 548 M*. tuberculosis* isolates recovered in 2013–2019 from 548 patients with pulmonary TB. All patients were newly diagnosed with TB, i.e. they have never had treatment for TB, or have taken anti-TB drugs for less than 1 month (https://www.ncbi.nlm.nih.gov/books/NBK138741/). All patients were permanent residents of the Omsk province in west Siberia, Russia, and were admitted to the Omsk Clinical tuberculosis dispensary.

According to the International Classification of Diseases 10^th^ revision (ICD-10, https://icd.who.int/browse10/2016/en), 542 patients in this study were classified as A.15 (respiratory tuberculosis, bacteriologically and histologically confirmed), 3 patients—A16.3 (tuberculosis of intrathoracic lymph nodes, without mention of bacteriological or histological confirmation), and 3 patients—A19 (miliary tuberculosis).

In addition, we considered the classification of clinical forms of tuberculosis officially used in Russia and approved by the Ministry of Health of the Russian Federation [20]. It is based on clinical, radiological and pathomorphological features of the TB process, its course and bacteriological confirmation. Thus in this study, the main clinical forms of TB were diagnosed as follows: infiltrative TB, fibrous-cavernous TB, disseminated TB of lungs, focal TB, and others. Infiltrative pulmonary TB is characterized by the presence of inflammatory foci in the lungs more than 1.0 cm in diameter, mostly exudative, with caseous necrosis and the presence or absence of lung tissue destruction and bronchogenic contamination. Fibrous-cavernous pulmonary TB is characterized by the presence of a fibrous cavity, the development of fibrotic changes, and other morphological changes in the lungs; the course is often accompanied by complications. Fibrous-cavernous TB indicates the long-term onset of the development of TB disease (several months) and non-effective chemotherapy. Disseminated TB of the lungs includes various processes that develop as a result of the spread of *M. tuberculosis* by hematogenous, lymphogenic, or mixed pathways; it occurs as acute, subacute or chronic. Focal pulmonary TB is characterized by the presence of focal formations up to 1.0 cm in diameter of productive, exudative, and caseous-necrotic genesis, localized in one or both lungs and occupying 1–2 segments.

Description of the sampling procedures is provided below and was also partly described previously [16, 21, 22]. The strain collection consisted of three groups as follows.

The first group included 97 MDR isolates from newly diagnosed patients from 2013 to 2015. These isolates were randomly selected among 228 MDR isolates recovered during the survey period [16]. The second group included 323 isolates obtained within a prospective study based on all consecutive newly diagnosed patients enrolled in 2015–2017; the first isolate from each second patient was enrolled in the study. The strategy of including every second isolate was based solely on resource availability. No additional selection criteria were applied and selection bias is unlikely to occur as inclusion was done in a consecutive manner. The groups of included and non-included cases did not differ significantly in any patient-related characteristics, such as gender, residence, age, diagnosis, HIV-coinfection. The third group included 128 isolates consecutively isolated from all newly diagnosed patients in 2019.

Ethical approval. This study was approved by the Ethical Committee of the Omsk State Medical University (protocols #66 of January 30, 2015, and #101 of February 8, 2018). All patients gave informed written consent to participate in this study.

*M. tuberculosis* drug susceptibility testing for the 1^st^ and 2^nd^ line drugs (streptomycin, isoniazid, rifampicin, ethambutol, pyrazinamide, ofloxacin, kanamycin, capreomycin, cycloserine; *para*-aminosalicylic acid) was carried out for all strains using the method of absolute concentrations on the solid Loewenstein-Jensen medium according to Order #109 of Ministry Healthcare of the Russian Federation and using Bactec MGIT 960 system (Becton Dickinson, Sparks, Md.) according to the manufacturer instructions. The bacteriology laboratory in the Omsk Clinical tuberculosis dispensary is externally quality assured by the System for External Quality Assessment "Center for External Quality Control of Clinical Laboratory Research" (Moscow, Russia).

Strains resistant to at least rifampin and isoniazid were defined as multidrug-resistant (MDR).

A number of the previously described molecular markers and tests was used to detect the Beijing genotype and its subtypes B0/W148 and Central Asian/Russian [11, 23, 24]. Non-Beijing strains were subjected to spoligotyping followed by the comparison with the SITVIT2 database [25]. Ancient sublineage of the Beijing genotype was detected using analysis of the NTF locus [26] and RD181 deletion [27]. Ancient Beijing sublineage strains were subjected to the 24 loci MIRU-VNTR typing followed by the comparison with MIRU-VNTRplus.org online tool and this permitted to detect two clusters 1071–32 and 14717–15. The LAM family was additionally tested by the detection of the specific SNP in the *Rv0129c* gene [28].

Statistical analysis was performed using Statistica 6.0 package (Statsoft Inc.) and MedCalc online tool (https://www.medcalc.org/calc/odds_ratio.php). A chi-square test was used to detect any significant difference between the two groups. Yates corrected χ2 and *P*-values were calculated with a 95% confidence interval (CI) around the mean. The significance threshold was set at *P* = 0.05.

## Results

In total, 548 M*. tuberculosis* isolates from 548 newly-diagnosed adult patients were included in the analysis. All patients were permanent residents of the Omsk province. Patients were stratified by age group, gender, residence (urban or rural). Clinical information included HIV coinfection, clinical forms of tuberculosis, and lethal outcome due to tuberculosis. *M. tuberculosis* strain information included phenotypic DST and strain genotype or subtype (in case of Beijing genotype).

The clinical forms of tuberculosis were diagnosed in the following numbers of patients: fibrous-cavernous TB (*n* = 33; 6.1%), infiltrative TB (*n* = 384; 70.0%), disseminated TB of the lungs (*n* = 82; 15.0%), focal TB of the lungs (11; 2.0%), and other (*n* = 38; 6.9%). Lethal outcome due to TB was recorded in 145 (26.5%) patients within one year after initial diagnosis.

Information on the distribution of genotypes among different groups by gender, age, clinical diagnosis, HIV status, lethal outcome, and DST is shown in Table 1. Almost 70% of the studied collection were male and 69% were in the age group of 18 to 44 years old. Infiltrative TB of the lungs was the main diagnosis that was found in a similar proportion (~ 70%) in almost all genotype groups.

**Table 1:** Characteristics of the patient subgroups infected with different *M. tuberculosis* genotypes

**Characteristics**	**Beijing** N (%)	**Modern Beijing**		**Ancient Beijing**				**non- Beijing** N (%)	**TOTAL** N (%)
		**B0/W148-cluster**	**Central Asian Russian**	**1071–32-cluster**	**14717–15-cluster**				
Total	370 (67.5%)	102 (18.6%)	219 (40.0%)	37 (6.7%)		12 (2.2%)		178 (32.5%)	548 (100%)
Gender									
Male	265 (71.7%)	77 (75.5%)	151 (68.9%)	28 (75.7%)		9 (75.0%)		128 (71.9%)	393 (71.7%)
Female	105 (28.3%)	25 (24.5%)	68 (31.1%)	9 (24.3%)		3 (25.0%)		50 (28.1%)	155 (28.3%)
Age, years old									
18–34	139 (37.6%)	42 (41.2%)	78 (35.6%)	14 (37.8%)		5 (41.7%)		60 (33.7%)	199 (36.3%)
35–44	128 (34.6%)	36 (35.3%)	73 (33.3%)	13 (35.2%)		5 (41.7%)		51 (28.7%)	179 (32.7%)
45–54	51 (13.8%)	13 (12.7%)	33 (15.1%)	6 (16.2%)		0		23 (12.9%)	74 (13.5%)
≥ 55	52 (14.0%)	11 (10.8%)	35 (16.0%)	4 (10.8%)		2		44 (24.7%)	96 (17.5%)
Urban	211 (57.0%)	62 (60.8%)	122 (55.7%)	20 (54.0%)		6 (50.0%)		100 (56.2%)	311 (56.7%)
Rural	159 (43.0%)	40 (39.2%)	96 (44.3%)	17 (46.0%)		6 (50.0%)		78 (43.8%)	237 (43.2%)
HIV-positive	143 (38.6%)	39 (38.2%)	83 (37.9%)	16 (43.2%)		5 (41.7%)		49 (27.5%)	192 (35.0%)
HIV-negative	227 (61.4%)	63 (62.8%)	136 (62.1%)	21 (56.8%)		7 (58.3%)		129 (72.5%)	356 (65.0%)
Clinical forms of tuberculosis (based on clinical and X-ray characteristics)									
Focal TB of lungs	6 (1.6%)	3 (2.9%)	2 (0.9%)	0		1 (8.3%)		5 (2.8%)	11 (2.0%)
Infiltrative TB of lungs	258 ( 69.7%)	68 (66.7%)	157 (71.7%)	22 (59.5%)		11 (91.7%)		126 (70.8%)	384 (70.0%)
Fibrous-cavernous TB	22 (5.9%)	8 (7.9%)	9 (4.1%)	5 (13.5%)		0		11 (6.2%)	33 (6.1%)
Disseminated TB of lungs	56 (15.1%)	16 (15.7%)	34 (15.5%)	6 (16.2%)		0		26 (14.6%)	82 (15.0%)
Other	27 (7.6%)	7 (6.8%)	17 (7.8%)	4 (10.8%)		0	10 (5.6%)		38 (6.9%)
*M. tuberculosis* drug resistance									
MDR	209 (56.5%)	92 (90.2%)	72 (32.9%)	37 (100%)		12 (100%)	38 (21.4%)		247 (45.1%)
Other resistance	47 (12.7%)	10 (9.8%)	37 (16.9%)	0		0	22 (12.3%)		69 (12.6%)
Susceptible	114 (30.8%)	0	110 (50.2%)	0		0	118 (66.3%)		232 (42.3%)
Lethal outcome due to TB	118 (31.9%)	32 (31.4%)	65 (29.7%)	14 (37.8%)		7 (58.3%)	27 (15.2%)		145 (26.5%)

The Beijing genotype was detected in 370 (67.5%) of the studied strains. The non-Beijing genotypes were detected in 178 (32.5%) isolates and they all belonged to the Euro-American lineage (Lineage 4): LAM – 61, ill-defined T – 56 (of them SIT53 – 21), Ural – 26, Haarlem – 17, unknown and other – 18 strains. The key findings on the non-Beijing genotypes are low 15% lethal outcome and compared to the Beijing genotype, lower MDR rate (21.4% vs 56.5%), and lower prevalence of HIV (27.5% vs 38.6%).

Given the major role of the dominant Beijing genotype in Russia, we further compared its different variants between them and against the non-Beijing genotypes pooled together. The Beijing genotype was represented by four genetic clusters: B0/W148 and Central Asian/Russian (both – modern sublineage), and 1071–32 and 14717–15 (both—ancient sublineage).

HIV rate was higher in Beijing compared to the non-Beijing group (38.6% vs 27.5%; *P* = 0.01; Odds ratio 1.66; 95% CI: 1.123 to 2.45). HIV rate was slightly higher among ancient compared to modern Beijing subtypes but non-significantly (43% vs 38%; *P* = 0.5).

Within the Beijing genotype, the most prevalent was Beijing Central Asian/Russian clade that was twice as prevalent as B0/W148 (40.0% vs 18.6%). Ancient Beijing clusters were detected in 8.9% of the total collection.

A major difference between genotypes and subtypes (Beijing clusters) in multidrug resistance and the lethal outcome was observed under some comparisons (Table 2, Supporting Tables S1-S6). A very high proportion of MDR isolates (90–100%) was a feature of the three of four Beijing clusters and, remarkably, it was the highest in both ancient clusters (100%). This is in contrast with the proportion of MDR isolates in the Beijing Central Asian/Russian clade (32.9%) and non-Beijing genotypes pooled together (21.4%).

**Table 2:** Pairwise comparisons of the proportion of MDR isolates between different Beijing clusters

	B0/W148 cluster	Central Asian Russian	B0/W148 cluster	1071–32	B0/W148 cluster	14717–15	Central Asian Russian	1071–32	Central Asian Russian	14717–15	1071–32	14717–15
MDR	92	72	92	37	92	12	72	37	72	12	37	12
Other	10	147	10	0	10	0	147	0	147	0	0	0
**χ**^**2**^	91.497		3.909		1.29		58.330		22.151		NaN	
***p***	< 0.001		0.049		0.257		< 0.001		< 0.001		1.0	

The group infected with 14717–15-cluster had the highest lethal outcome (58.3%) compared to the groups infected with Russian epidemic strain Beijing B0/W148 (31.4%, *P* = 0.06), Beijing Central Asian/Russian clade (29.7%, *P* = 0.037), and non-Beijing strains (15.2%, *P* = 0.001). Two major clusters of the modern Beijing (B0/W148 and Central Asian/Russian) did not differ in the lethal outcome although they did differ in the MDR rate.

The percent of fibrous-cavernous TB was the highest in the 1071–32 group (13.5%) compared to 0% in the 14717–15 group. Infiltrative TB was detected at the highest rate in the 14717–15 group (91.7%) compared to the lowest rate in the 1071–32 group (59.5%, *P* = 0.06) and lower rate in patients infected with modern Beijing clusters B0/W148 and Central Asian/Russian together (70.1%; *P* = 0.1). Disseminated TB of the lungs did not correlate in this study with any particular *M. tuberculosis* genotype (mostly being at 15–16% in Beijing and non-Beijing alike). The only exception was the 14717–15 cluster group that did not include such patients.

With regard to age, some trends albeit non-significant are noteworthy. Patients infected with major modern Beijing subtypes are dominated by patients aged < 44 years old but this percent is slightly higher in the B0/W148 group – 76.5% compared to 68.9% of the Central Asian/Russian clade. In contrast, older patients (> 55 years old) were more prevalent in the Central Asian/Russian group compared to the B0/W148-cluster group: 16.0% vs 10.8%. A small sample size of the 14717–15 group (*n* = 12) precludes from drawing robust conclusions nevertheless we note that 10 of 12 patients were < 44 years old (two other patients were 55 and 58 years old), thus this strain appears to be marked with an ongoing circulation and likely recent transmission.

## Discussion

*M. tuberculosis* population in Russia is dominated by the Beijing genotype which major epidemic and endemic variants are characterized by contrasting drug resistance and virulence. Here, we studied how *M. tuberculosis* strain pathobiology could influence the progression of tuberculosis manifested through clinical features and the lethal outcome of the disease.

Previous studies carried out in different settings worldwide reported adverse outcomes of the TB caused by the Beijing genotype strains that were manifested by the febrile response, severe intoxication, a combination of pulmonary and extrapulmonary disease, MDR, and XDR TB, and generalized disease [2, 29–32]. Extrathoracic involvement was three-time more frequent in patients infected with Beijing isolates [2]. Strains of the East Asian lineage (i.e. mostly Beijing genotype) were more capable of extra-pulmonary dissemination and TB meningitis compared to Euro-American lineage in Vietnam [30]. A hypervirulent *M. tuberculosis* Beijing strain exhibited significantly better intracellular survivability and induced a lower level of pro-inflammatory TNF-α than the reference virulent strain H37Rv in the human macrophage challenge experiment [33]. That Beijing strain also had certain mutations in the virulence-associated loci *Rv0178* within *mce1* operon and an intergenic deletion near *phoP* although the causative role of these mutations in mycobacterial virulence is yet to be validated. In the WGS based study in Peru, mutations *Rv2828c*.141 and *rpoC*.1040 were significantly associated with more widespread radiological pathology [34].

At the same time, the occasional lack of association between lineage and severity (e.g., [34]), could be due to pooling together distant clusters within the same but heterogeneous lineage. This highlights the particular importance of analyzing neither large lineages nor single individual strains but rather middle-sized clusters of the genetically related isolates that perhaps may be defined as the major epidemiological “actors” on the *M. tuberculosis* side.

In Russian settings, the Beijing genotype on the whole was frequently associated with disseminated, fibrous-cavernous forms of tuberculosis, as well as caseous pneumonia [35]. Chronic patients with fibrous-cavernous TB were frequently infected with Beijing B0/W148 strain in Irkutsk, East Siberia and those receiving 3–4 courses of chemotherapy had an even higher rate of this genotype (66.7% vs 43.7%) [36]. The same study showed that disseminated TB was more frequently diagnosed in the B0/W148 group compared to other genotypes (37.5% vs 15.7%), which was associated with a high rate of HIV coinfection (56.3%).

In this study, the genotypes and respective patient subgroups were compared in the rate of MDR, HIV coinfection, lethal outcome, patient’s age, clinical forms of pulmonary TB. High lethality/virulence in the mouse model previously demonstrated for Beijing 14717–15-cluster [18] corresponded to the increased rate of lethal outcomes in patients infected with this strain cluster.

Disseminated TB of lungs was previously correlated with the higher rate of HIV coinfection and Beijing B0/W148 strain [36] but in this study, it did not correlate with a particular genotype, furthermore without difference between Beijing and non-Beijing. However, one remarkable exception should be noted: no patients with disseminated TB of lungs were found in the group infected with highly virulent and lethal Beijing 14717–15 cluster (0/12, compared to 50/321 [15.6%] for modern Beijing clusters B0/W148 and Central Asian/Russian together; *P* = 0.3). This may be interpreted as a reflection of the particular features of this 14717–15 cluster demonstrated in the murine model (more pronounced weight loss, higher bacterial burden, and more severe lung pathology) and under in vitro growth study (significantly shorter lag-phase) [18].

The chronicity of TB may be reflected by the development of the fibrous-cavernous disease. The percent of fibrous-cavernous TB was the highest in the 1071–32-cluster group (13.5%) likely because this cluster was low virulent in the murine model [18], compared to 0% of fibrous-cavernous TB in patients infected with hypervirulent 14717–15-cluster. Chronicity may be explained by low virulence/lethality of the 1071–32 strain and along with its MDR/XDR association can lead to strain resistance to chemotherapy and consequently prolonged treatment.

## Conclusions

In spite of the earlier views, the *M. tuberculosis* Beijing genotype is heterogeneous both genetically and pathobiologically even in the area of its active ongoing circulation and expected reduced diversity, i.e., Russia. As a side conclusion, this underlines the importance of using not single but multiple and genetically diverse Beijing strains in different model experiments. In this study, the Beijing genotype as a whole was associated with an MDR phenotype but members of some Beijing subtypes were significantly more likely to be MDR. These were well-known Russian epidemic B0/W148, but also two emerging clusters 1071–32 and 14717–15 of the ancient Beijing sublineage that were exclusively MDR. This is in contrast with the MDR rate in the other Beijing cluster Central Asian/Russian (32.9%) and non-Beijing genotypes pooled together (21.4%).

This study indirectly shows that the traditional approach to assessing virulence and lethality in murine models does remain useful. The group infected with hypervirulent and highly lethal in murine model 14717–15 cluster had the highest rate of the lethal outcome (58.3%) compared to Beijing B0/W148 (31.4%), and non-Beijing (15.2%) groups. The 14717–15 cluster mostly included isolates from patients with infiltrative but not with fibrous-cavernous and disseminated TB. In contrast, the patient group infected with another ancient Beijing cluster, low virulent MDR 1071–32 had the highest rate of fibrous-cavernous TB, likely reflecting the capacity of these strains of prolonged survival and chronicity of the TB process.

Subgroups of patients infected with the modern Beijing sublineage and ancient 14717–15 cluster were dominated by patients aged < 44 years which likely correlates with active ongoing transmission of these strains. Keeping in mind that disseminated disease may take longer to manifest, a recent transmission of Beijing 14717–15 could speculatively be linked to the lack of disseminated disease among patients infected with this subtype.

This study highlights the importance of continuous molecular surveillance of existing and newly emerging strains to detect epidemiological trends on the whole and inform treatment of individual patients in particular. In Russia, a country with a very high rate of primary MDR-TB, treatment is empirical and takes into account a high probability of primary MDR-TB for some genotypes. Now it is time to attempt considering other features of the infecting strains as well. Not only drug resistance but also strain virulence should be taken into consideration in personalized medicine and TB treatment.

## Supplementary Information

**Additional file 1: Table S1.** Comparison of Beijing B0/W148 and Central Asian / Russian clusters. **Table S2.** Comparison of Beijing B0/W148 and 1071-32 clusters. **Table S3.** Comparison of Beijing B0/W148 and 14717-15 clusters. **Table S4.** Comparison of the Beijing Central Asian Russian and 1071-32 clusters. **Table S5.** Comparison of Beijing Central Asian Russian and 14717-15 clusters. **Table S6.** Comparison of Beijing 1071-32 and 14717-15 clusters.

## Data Availability

The datasets generated and/or analysed during the current study are not publicly available due to limitations of ethical approval involving the patient data and anonymity but are available from the corresponding author on reasonable request.

## Abbreviations

TB: Tuberculosis
MDR: Multidrug resistant
XDR: Extensively drug resistant
MIRU-VNTR: Mycobacterial interspersed repetitive units—variable number of tandem repeats
DST: Drug susceptibility testing
