# Supplementary material for: Impact of pathobiological diversity of Mycobacterium tuberculosis on clinical features and lethal outcome of tuberculosis
Source: BMC Microbiol. 2022 Feb 8;22:50. doi: 10.1186/s12866-022-02461-w (PMC8822639; doi:10.1186/s12866-022-02461-w)
Supplement: Supplementary file 1 — Additional file 1: Table S1. Comparison of Beijing B0/W148 and Central Asian / Russian clusters. Table S2. Comparison of Beijing B0/W148 and 1071-32 clusters. Table S3. Comparison of Beijing B0/W148 and 14717-15 clusters. Table S4. Comparison of the Beijing Central Asian Russian and 1071-32 clusters. Table S5. Comparison of Beijing Central Asian Russian and 14717-15 clusters. Table S6. Comparison of Beijing 1071-32 and 14717-15 clusters. [file 12866_2022_2461_MOESM1_ESM.docx]

BMC Microbiology

Igor Mokrousov, Oksana Pasechnik, Anna Vyazovaya, Irina Yarusova, Alena Gerasimova, Aleksey Blokh, Viacheslav Zhuravlev

**Impact of pathobiological diversity of *Mycobacterium tuberculosis* on clinical features and lethal outcome of tuberculosis**

**Additional file 1**

**Table S1.** Comparison of Beijing B0/W148 and Central Asian / Russian clusters

| **Characteristics** | **Beijing cluster** | | **χ^2^** | **р** |
| --- | --- | --- | --- | --- |
|  | **B0/W148** | **Central Asian Russian** |  |  |
| Total | 102 | 219 |  |  |
| Gender | | | | |
| Man | 77 | 151 | 1.447 | 0.23 |
| Woman | 25 | 68 |  |  |
| Age. years old | | | | |
| 18-34 | 42 | 78 | 2.228 | 0.527 |
| 35-44 | 36 | 73 |  |  |
| 45-54 | 13 | 33 |  |  |
| ≥55 | 11 | 35 |  |  |
| Place of residence | | | | |
| Urban | 62 | 122 | 0.661 | 0.417 |
| Rural | 40 | 96 |  |  |
| HIV-status | | | | |
| HIV-positive | 39 | 83 | 0.003 | 0.954 |
| HIV-negative | 63 | 136 |  |  |
| Clinical forms of tuberculosis (based on clinical and X-ray characteristics) | |  |  |  |
| Focal TB of lungs | 3 | 2 | 4.433 | >0.05 |
| infiltrative TB of lungs | 68 | 157 |  |  |
| fibrous-cavernous TB | 8 | 9 |  |  |
| disseminated TB of lungs | 16 | 34 |  |  |
| Other | 7 | 17 |  |  |
| *M. tuberculosis* drug resistance | | | | |
| MDR | 92 | 72 | **98.372** | **<0.001** |
| Other resistance | 10 | 37 |  |  |
| Susceptible to all tested drugs | 0 | 110 |  |  |
| Lethal outcome due to TB | 32 | 65 | 0.094 | 0.759 |

**Table S2.** Comparison of Beijing B0/W148 and 1071-32 clusters

| **Characteristics** | **Beijing cluster** | | **χ^2^** | **р** |
| --- | --- | --- | --- | --- |
|  | **B0/W148** | **1071-32** |  |  |
| Total | 102 | 37 |  |  |
| Gender | | | | |
| Man | 77 | 28 | 0.001 | 0.983 |
| Woman | 25 | 9 |  |  |
| Age. years old | | | | |
| 18-34 | 42 | 14 | 0.315 | 0.958 |
| 35-44 | 36 | 13 |  |  |
| 45-54 | 13 | 6 |  |  |
| ≥55 | 11 | 4 |  |  |
| Place of residence | | | | |
| Urban | 62 | 20 | 0.508 | 0.476 |
| Rural | 40 | 17 |  |  |
| HIV-status | | | | |
| HIV-positive | 39 | 16 | 0.285 | 0.594 |
| HIV-negative | 63 | 21 |  |  |
| Clinical forms of tuberculosis (based on clinical and X-ray characteristics) | | | | |
| Focal TB of lungs | 3 | 0 | 5.720 | >0.05 |
| infiltrative TB of lungs | 68 | 22 |  |  |
| fibrous-cavernous TB | 8 | 5 |  |  |
| disseminated TB of lungs | 16 | 2 |  |  |
| Other | 7 | 4 |  |  |
| *M. tuberculosis* drug resistance | | | | |
| MDR | 92 | 37 | 3.909 | 0.049 |
| Other resistance | 10 | 0 |  |  |
| Susceptible to all tested drugs | 0 | 0 |  |  |
| Lethal outcome due to TB | 32 | 14 | 0.513 | 0.475 |

**Table S3.** Comparison of Beijing B0/W148 and 14717-15 clusters

| **Characteristics** | **Beijing cluster** | | **χ^2^** | **р** |
| --- | --- | --- | --- | --- |
|  | **B0/W148** | **14717-15** |  |  |
| Total | 102 | 12 |  |  |
| Gender | | | | |
| Man | 77 | 9 | 0.001 | 0.971 |
| Woman | 25 | 3 |  |  |
| Age. years old | | | | |
| 18-34 | 42 | 5 | 1.977 | 0.578 |
| 35-44 | 36 | 5 |  |  |
| 45-54 | 13 | 0 |  |  |
| ≥55 | 11 | 2 |  |  |
| Place of residence | | | | |
| Urban | 62 | 6 | 0.519 | 0.472 |
| Rural | 40 | 6 |  |  |
| HIV-status | | | | |
| HIV-positive | 39 | 5 | 0.053 | 0.818 |
| HIV-negative | 63 | 7 |  |  |
| Clinical forms of tuberculosis (based on clinical and X-ray characteristics) | | | | |
| Focal TB of lungs | 3 | 1 | 3.516 | >0.05 |
| infiltrative TB of lungs | 68 | 11 |  |  |
| fibrous-cavernous TB | 8 | 0 |  |  |
| disseminated TB of lungs | 16 | 4 |  |  |
| Other | 7 | 0 |  |  |
| *M. tuberculosis* drug resistance | | | | |
| MDR | 92 | 12 | 1.29 | 0.257 |
| Other resistance | 10 | 0 |  |  |
| Susceptible to all tested drugs | 0 | 0 |  |  |
| Lethal outcome due to TB | 32 | 7 | 3.468 | 0.063 |

**Table S4.**

Comparison of the Beijing Central Asian Russian and 1071-32 clusters

| **Characteristics** | **Beijing cluster** | | **χ^2^** | **р** |
| --- | --- | --- | --- | --- |
|  | **Central Asian Russian** | **1071-32** |  |  |
| Total | 219 | 37 |  |  |
| Gender | | | | |
| Man | 151 | 28 | 0.681 | 0.410 |
| Woman | 68 | 9 |  |  |
| Age. years old | | | | |
| 18-34 | 78 | 14 | 0.657 | 0.884 |
| 35-44 | 73 | 13 |  |  |
| 45-54 | 33 | 6 |  |  |
| ≥55 | 35 | 4 |  |  |
| Place of residence | | | | |
| Urban | 122 | 20 | 0.047 | 0.829 |
| Rural | 96 | 17 |  |  |
| HIV-status | | | | |
| HIV-positive | 83 | 16 | 0.381 | 0.538 |
| HIV-negative | 136 | 21 |  |  |
| Clinical forms of tuberculosis (based on clinical and X-ray characteristics) | | | | |
| Focal TB of lungs | 2 | 0 | 9.484 | >0.05 |
| infiltrative TB of lungs | 157 | 22 |  |  |
| fibrous-cavernous TB | 9 | 5 |  |  |
| disseminated TB of lungs | 34 | 2 |  |  |
| Other | 17 | 4 |  |  |
| *M. tuberculosis* drug resistance | | | | |
| MDR | 72 | 37 | 58.330 | <0.001 |
| Other resistance | 37 | 0 |  |  |
| Susceptible to all tested drugs | 110 | 0 |  |  |
| Lethal outcome due to TB | 65 | 14 | 0.987 | 0.321 |

**Table S5.** Comparison of Beijing Central Asian Russian and 14717-15 clusters

| **Characteristics** | **Beijing cluster** | | **χ^2^** | **р** |
| --- | --- | --- | --- | --- |
|  | **Central Asian Russian** | **14717-15** |  |  |
| Total | 219 | 12 |  |  |
| Gender | | | | |
| Man | 151 | 9 | 0.196 | 0.659 |
| Woman | 68 | 3 |  |  |
| Age. years old | | | | |
| 18-34 | 78 | 5 | 2.161 | 0.540 |
| 35-44 | 73 | 5 |  |  |
| 45-54 | 33 | 0 |  |  |
| ≥55 | 35 | 2 |  |  |
| Place of residence | | | | |
| Urban | 122 | 6 | 0.164 | 0.686 |
| Rural | 96 | 6 |  |  |
| HIV-status | | | | |
| HIV-positive | 83 | 5 | 0.068 | 0.794 |
| HIV-negative | 136 | 7 |  |  |
| Clinical forms of tuberculosis (based on clinical and X-ray characteristics) | | | | |
| Focal TB of lungs | 2 | 1 | 6.072 | >0.05 |
| infiltrative TB of lungs | 157 | 11 |  |  |
| fibrous-cavernous TB | 9 | 0 |  |  |
| disseminated TB of lungs | 34 | 4 |  |  |
|  |  |  |  |  |
| Other | 17 | 0 |  |  |
| *M. tuberculosis* drug resistance | | | | |
| MDR | 72 | 12 | **22.151** | **0.001** |
| Other resistance | 37 | 0 |  |  |
| Susceptible to all tested drugs | 110 | 0 |  |  |
| Lethal outcome due to TB | 65 | 7 | **4.354** | **0.037** |

**Table S6.** Comparison of Beijing 1071-32 and 14717-15 clusters

| **Characteristics** | **Beijing cluster** | | **χ^2^** | **р** |
| --- | --- | --- | --- | --- |
|  | **1071-32** | **14717-15** |  |  |
| Total |  |  |  |  |
| Gender | | | | |
| Man | 28 | 9 | 0.002 | 0.963 |
| Woman | 9 | 3 |  |  |
| Age. years old | | | | |
| 18-34 | 14 | 5 | 2.339 | 0.506 |
| 35-44 | 13 | 5 |  |  |
| 45-54 | 6 | 0 |  |  |
| ≥55 | 4 | 2 |  |  |
| Place of residence | | | | |
| Urban | 20 | 6 | 0.06 | 0.807 |
| Rural | 17 | 6 |  |  |
| HIV-status | | | | |
| HIV-positive | 16 | 5 | 0.009 | 0.924 |
| HIV-negative | 21 | 7 |  |  |
| Clinical forms of tuberculosis (based on clinical and X-ray characteristics) | | | | |
| Focal TB of lungs | 0 | 1 | N | >0.05 |
| infiltrative TB of lungs | 22 | 11 |  |  |
| fibrous-cavernous TB | 5 | 0 |  |  |
| disseminated TB of lungs | 6 | 0 |  |  |
| Other | 4 | 0 |  |  |
| *M. tuberculosis* drug resistance | | | | |
| MDR | 37 | 12 | - | 1 |
| Other resistance | 0 | 0 |  |  |
| Susceptible to all tested drugs | 0 | 0 |  |  |
| Lethal outcome due to TB | 14 | 7 | 1.554 | 0.213 |
